# Thiazolides promote apoptosis in colorectal tumor cells via MAP kinase-induced Bim and Puma activation

**DOI:** 10.1038/cddis.2015.137

**Published:** 2015-06-04

**Authors:** A Brockmann, A Bluwstein, A Kögel, S May, A Marx, M P Tschan, T Brunner

**Affiliations:** 1 Chair of Biochemical Pharmacology, Department of Biology, University of Konstanz, Konstanz, Germany; 2 Konstanz Research School Chemical Biology, University of Konstanz, Konstanz, Germany; 3 Chair of Organic Chemistry/Cellular Chemistry, Department of Chemistry, University of Konstanz, Konstanz, Germany; 4 Division of Experimental Pathology, Institute of Pathology, University of Bern, Bern, Switzerland

## Abstract

While many anticancer therapies aim to target the death of tumor cells, sophisticated resistance mechanisms in the tumor cells prevent cell death induction. In particular enzymes of the glutathion-S-transferase (GST) family represent a well-known detoxification mechanism, which limit the effect of chemotherapeutic drugs in tumor cells. Specifically, GST of the class P1 (GSTP1-1) is overexpressed in colorectal tumor cells and renders them resistant to various drugs. Thus, GSTP1-1 has become an important therapeutic target. We have recently shown that thiazolides, a novel class of anti-infectious drugs, induce apoptosis in colorectal tumor cells in a GSTP1-1-dependent manner, thereby bypassing this GSTP1-1-mediated drug resistance. In this study we investigated in detail the underlying mechanism of thiazolide-induced apoptosis induction in colorectal tumor cells. Thiazolides induce the activation of p38 and Jun kinase, which is required for thiazolide-induced cell death. Activation of these MAP kinases results in increased expression of the pro-apoptotic Bcl-2 homologs Bim and Puma, which inducibly bind and sequester Mcl-1 and Bcl-x_L_ leading to the induction of the mitochondrial apoptosis pathway. Of interest, while an increase in intracellular glutathione levels resulted in increased resistance to cisplatin, it sensitized colorectal tumor cells to thiazolide-induced apoptosis by promoting increased Jun kinase activation and Bim induction. Thus, thiazolides may represent an interesting novel class of anti-tumor agents by specifically targeting tumor resistance mechanisms, such as GSTP1-1.

Glutathione-*S*-transferases (GSTs) represent a superfamily of cellular phase II detoxification enzyme. Specifically, GSTs catalyze the conjugation of electrophilic substances to the tripeptid glutathione (GSH, *γ*-L-glutamyl-L-cysteinylglycine). Thereby, hazardous metabolic products, xenobiotics and oxidative stress products become rapidly neutralized by GSTs, protecting cells from potentially damaging substances and carcinogens. Consequently, GSTs have a critical role in the detoxification of cells and inactivation of drugs.^1, 2^ At the present, seven classes of mammalian cytosolic GSTs are known, whose expression is regulated in a tissue-specific manner^3, 4, 5^ pointing toward a defined role of individual GSTs in the biotransformation of drugs and reactive compounds in diverse tissues.^6, 7^

GSTs have a critical role in tumor therapy, as numerous tumors overexpress various GSTs, which contribute to the development of resistance to chemotherapeutic treatments.^8, 9^ In particular, high expression levels of GST class pi (GSTP1-1) have been reported in a wide range of solid tumors, such as colon, breast, kidney, pancreas, lung, and ovarian cancer cells, and lymphoma.^10, 11, 12^ The sensitivity of these tumors toward chemotherapeutic drugs, such as cisplatin, doxorubicin, and etoposide, is negatively affected by high expression levels of GSTP1-1.^13, 14, 15, 16, 17^ Thus, overexpression of GSTP1-1 in solid tumors limits the therapeutic effects of different chemotherapeutic drugs via their GSTP1-1-mediated inactivation.

This observation identifies GSTs in general and GSTP1-1 in particular as important therapeutic targets in the treatment of solid tumors. Consequently, small molecular inhibitors of GSTs have been developed in the past to modulate GST activities and drug resistance of tumor cells, thereby sensitizing tumor cells to anticancer drugs. The therapeutic effect of the competitive inhibitors ethacrynic acid (EA) was proven in a clinical trial;^18^ however, long-term utility of EA was limited by its strong diuretic properties.^19^ A somewhat different approach includes the GST-activated pro-drugs and the GSH analog TLK199. TLK199 is metabolized and subsequently inhibits GST activities, making it a more selective GST inhibitor.^20^ However, thus far experimental and clinical data on solid tumors are missing.

Thiazolides are a novel class of anti-infectious drugs used for the treatment of intestinal infection, and show a broad activity against intestinal pathogens.^21, 22, 23, 24^ The parent compound nitazoxanide (NTZ; 2-(acetolyloxy)-*N*-(5-nitro-2-thiazolyl)benzamide) is successfully used for the treatment of *Giardia lamblia* and *Cryptosporidium parvum* infections.^25, 26, 27, 28^ Though thiazolides generally have minimal side effects on host tissue cells during therapeutic treatments,^29^ it was recently noticed that they promote apoptosis induction in colorectal tumor cells, however, sparing non-transformed cells.^30^ Of interest, while the bromo-thiazolide RM4819 (*N*-(5-bromothiazol-2-yl)2-hydroxy-3-methylbenzamide) shows only reduced anti-microbial activity, both NTZ and RM4819 promote cell death in colorectal tumor cells. This indicates that the therapeutic targets of thiazolides are substantially different in intestinal parasites and colorectal tumor cells. Subsequent studies identified GSTP1-1 as a major RM4819-binding partner in colorectal tumor cells.^30^ While it was initially thought that thiazolides are inhibitors of GSTP1-1, it is presently accepted that GSTP1-1 is required for thiazolide-induced cell death induction. Interestingly, an *N*-acetyl-L-cysteine (NAC)-induced increase in cellular GSH levels enhanced thiazolide-induced cell death, whereas it lowered the sensitivity toward chemotherapeutic drugs by promoting their GSTP1-1-mediated inactivation.^31^ Thus, thiazolides appear to represent a novel class of GSTP1-1-activated pro-drugs, activated likely by conjugation to GSH, rather than GSTP1-1 inhibitors. This makes thiazolides an interesting novel class of anti-tumor drugs specifically targeting tumors with elevated levels of GSTs, and GSTP1-1 an Achilles' heel for the potential therapeutic action of thiazolides. While thiazolides alone are relatively slow and weak inducers of apoptosis in colorectal tumor cells, they profoundly synergize with inducers of the intrinsic apoptosis pathway, such as chemotherapeutic drugs, as well triggers of the extrinsic pathway, such as TRAIL (TNF-related apoptosis-inducing ligand).^31^

The mechanism of thiazolide-induced apoptosis and sensitization of tumor cells to other apoptosis triggers is presently incompletely understood, although GSTP1-1, the activation of the MAP kinases, and the Bcl-2-regulated mitochondrial apoptosis pathways appear to have a critical role in this process.^31^ In this study we investigated in more detail the underlying molecular signaling pathways leading to thiazolide-induced cell death in colorectal tumor cells. We find that activity of both the MAP kinases p38 and Jun kinase (JNK) is critical for mediating thiazolide-induced apoptosis, as their combined inhibition blocks cell death induction. In particular JNK was found to be important for the induction and activation of the downstream effectors of the Bcl-2 family, that is, the BH3-only proteins Bim and Puma. Bim and Puma appear to activate the mitochondrial pathway by interacting with and neutralizing the anti-apoptotic Bcl-2 homolog Bcl-x_L_, and inhibition of JNK prevented Bim and Puma induction, interaction with Bcl-x_L_, and induction of apoptosis. Furthermore, thiazolides induced interaction of Bim with Mcl-1 and promote the degradation of Mcl-1. While an increase in cellular GSH levels inhibited chemotherapy-induced apoptosis, it resulted in a more robust activation of JNK, Bim induction, Mcl-1 degradation, and associated thiazolide-induced cell death.

In summary, we here show that thiazolides are a novel group of GSTP1-1-activated pro-drugs, which activate the mitochondrial apoptosis pathway at different levels. Given that GSTs are highly overexpressed in numerous tumors and that GSTs contribute to therapy resistance of these tumors, thiazolides may become an interesting therapeutic option for the treatment of chemoresistant tumor cells.

## Results

### Thiazolides induce JNK- and p38-dependent cell death

**Figure 1:**
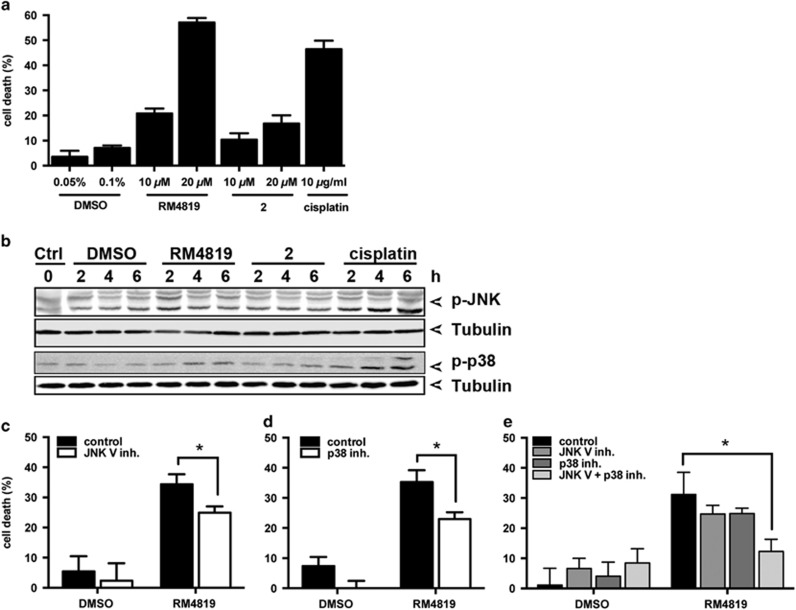
Thiazolide-induced cell death is JNK and p38-dependent. (**a**) Caco-2 cells were treated with indicated concentrations of RM4819, compound 2, cisplatin, or DMSO as solvent control for 40 h. Cell death induction was calculated by an MTT assay. Mean values of triplicates±S.D. of a representative experiment are shown (*n*>3). (**b**) Caco-2 cells were control treated (ctrl), or with thiazolides (20 *μ*M), cisplatin (10 *μ*g/ml), or DMSO (0.1%) for indicated time intervals. Phospho-JNK (p-JNK), phospho-p38 (p-p38), and tubulin as a loading control were detected by western blotting. (**c–e)** Caco-2 cells were pretreated with JNK V inhibitor (2.5 *μ*M) (**c**), p38 inhibitor (10 *μ*M), (**d**) or the combination of both (**e**) 1 h before stimulation with 20 *μ*M RM4819 for further 40 h. Cell death induction was assessed by an MTT assay. Mean values of triplicates±S.D. of a representative experiment are shown (*n*>3). *P<0.01

The molecular structure of thiazolides consists of a thiazole-ring and a benzene ring linked by an amide bond. We have previously performed a structure-function study and shown that changes of substituents in the benzene ring do not affect the cell death-promoting activity of thiazolides, whereas removal of the bromide atom from the thiazole ring, as in compound 2, strongly reduces the activity.^32^ To investigate the thiazolide-induced apoptosis signaling pathways, we employed RM4819 as an active thiazolide, and compound 2 is inactive control substance to induce apoptosis in the colorectal tumor cell lines Caco-2 and LS174T. Figure 1a (Caco-2 cells) and Supplementary Figure 1 (LS174T cells) show that RM4819 induced cell death in a dose-dependent manner, whereas cells were almost insensitive to compound 2. In contrast, the chemotherapeutic drug cisplatin promoted cell death in both colorectal tumor cell lines.

Next, the ability of RM4819 and compound 2 in inducing activation of the MAP kinases JNK and p38 was assessed. JNK activation was observed at around 2 h and p38 activation 4 h after stimulation with RM4819. As expected from its lack of apoptosis-promoting activity stimulation of cells with compound 2 did not result in a detectable increase in MAP kinase activation (Figure 1b). While RM4819-induced MAP kinase activation was specific, though relatively slow and weak, the control substance cisplatin induced a pronounced and sustained activation of both, JNK and p38 (Figure 1b).

To address the relevance of JNK and p38 activation in thiazolide-induced cell death, we employed pharmacological inhibitors of JNK and p38. Interestingly, while inhibition of either JNK or p38 only partially blocked RM4819-induced apoptosis, only combined inhibition of both, JNK and p38 almost completely prevented cell death induction. This indicates that both MAP kinases are involved in thiazolide-induced cell death in Caco-2 cells.

### Thiazolides modulate expression levels of Bcl-2 family members

**Figure 2:**
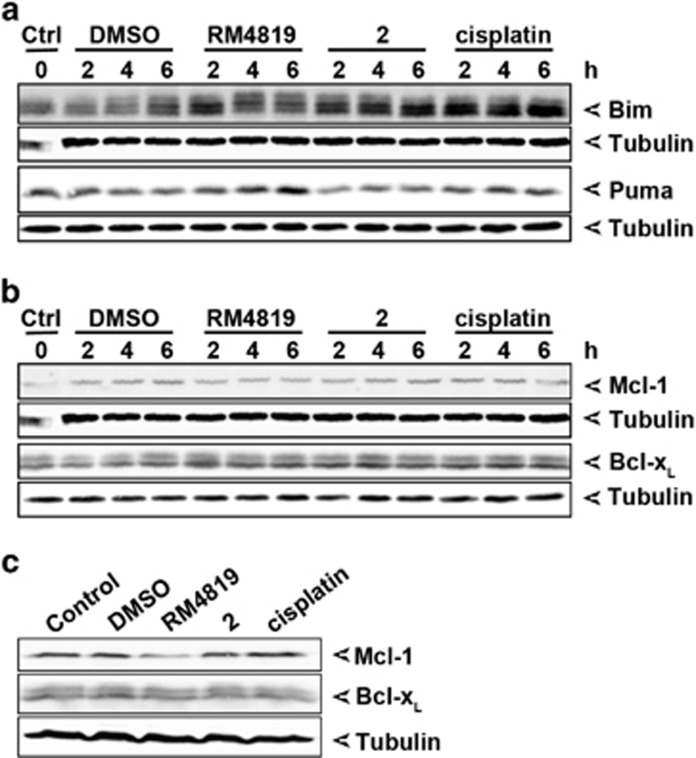
Changes in thiazolide-induced Bcl-2 family member expression. (**a** and **b**) Caco-2 cells were treated with 20 *μ*M thiazolides, 10 *μ*g/ml cisplatin, or 0.1% DMSO for 2, 4, and 6 h, and Bim and Puma (**a**), Mcl-1 and Bcl-x_L_ (**b**) expression levels were detected by western blot. (**c**) Mcl-1 and Bcl-x_L_ expression levels were detected 8 h after treatment with complete medium as control, 0.1% DMSO, 20 *μ*M RM4819 or compound 2, or 10 *μ*g/ml cisplatin. Tubulin served as a loading control

We have previously shown that thiazolides induce apoptosis via the Bcl-2 family-orchestrated mitochondrial apoptosis pathway. Specifically, we have seen that thiazolides induce the expression of Bim, which participates in thiazolide-induced cell death.^31^ But likely other components of the mitochondrial apoptosis pathway contribute to the execution of thiazolide-induced apoptosis. To understand whether and how thiazolides affect expression levels of different pro- and anti-apoptotic Bcl-2 homologs, cells were stimulated with RM4819, compound 2, and cisplatin as control, and levels of Bim, Puma, Bcl-x_L_, and Mcl-1 were assessed by western blotting. As found previously, we observed an RM4819-induced increase in Bim levels already 2 h after stimulation. Of interest, after 4 and 6 h Bim levels were not further induced but showed an increase in higher molecular weight species of Bim_EL_, indicative of Bim phosphorylation.^33, 34, 35^ In marked contrast, only a minimal increase in Bim expression over that of control treated cells was observed in response to compound 2, with no apparent induction of Bim phosphorylation. Cisplatin treatment resulted in a strong increase in Bim expression, but not Bim phosphorylation. RM4819, but not compound 2 and cisplatin, also induced a substantial increase in Puma expression (Figure 2a and Supplementary Figure 2). While Bcl-x_L_ levels remained unaffected by the treatment of cells with the different substances, a decrease of Mcl-1 expression was noticed after stimulation of the cells with RM4819, but not with compound 2 or cisplatin (Figures 2b and c). This finding was also confirmed in other colorectal tumor cells (LS174T) and for later time points (16 h) (Supplementary Figures 2a and b). This RM4819-induced degradation of Mcl-1 is likely not a consequence of caspase activation, as the pan-caspase inhibitor zVAD failed to prevent Mcl-1 degradation, whereas the proteosome inhibitor MG132 stabilized it (Supplementary Figure 2c).

### RM4819-induced apoptosis is dependent on Bim and Puma

**Figure 3:**
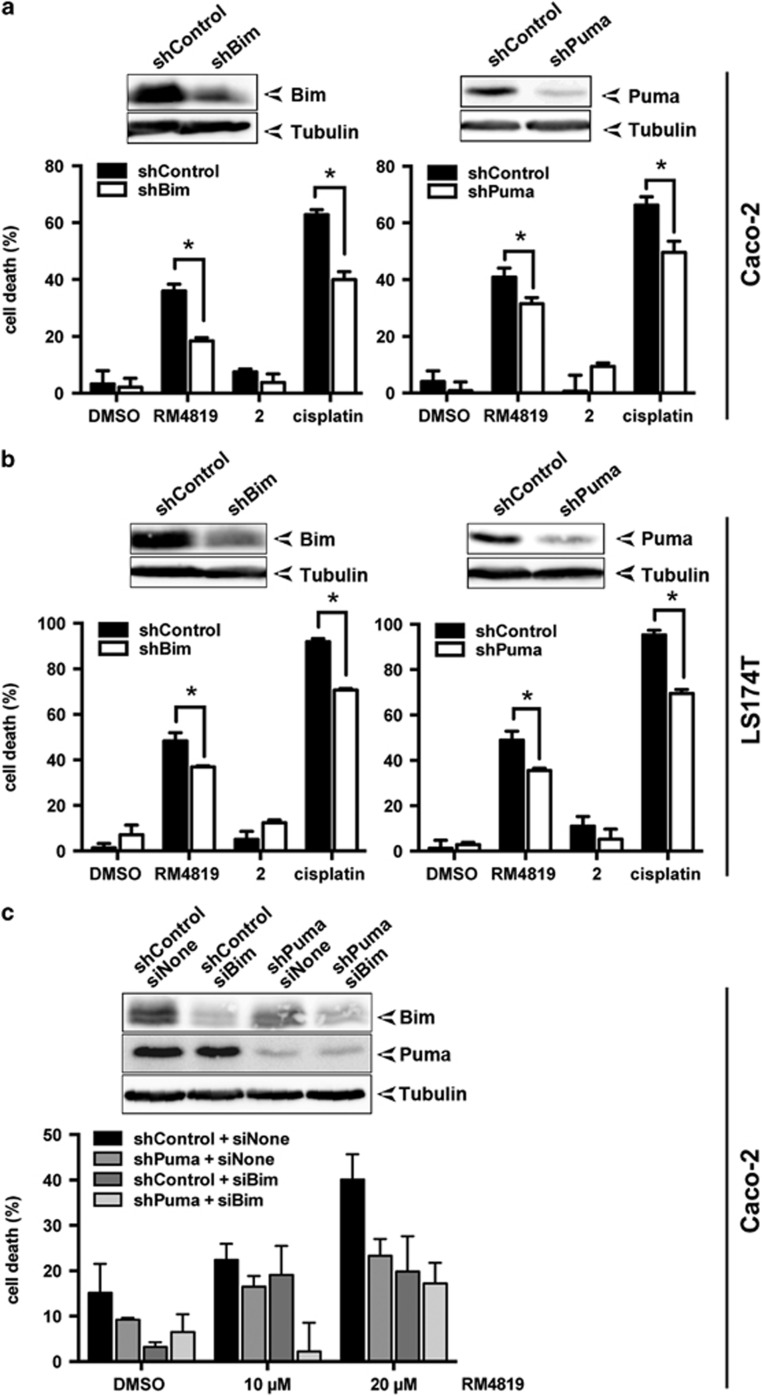
Thiazolide-induced cell death is regulated by Bim and Puma. Caco-2 (**a**) and LS174T (**b**) cells were transduced with Bim- or Puma-targeting shRNA (shBim, shPuma) or non-targeting shRNA (shControl). Bim and Puma knockdown efficiency was monitored by western blot (insets). Cells transduced with shControl, shBim, or shPuma were treated with DMSO (0.1%), RM4819 (20 *μ*M), or cisplatin (1 *μ*g/ml) for 40 h. Cell death induction was calculated by an MTT assay. Mean values of triplicates±S.D. of a representative experiment are shown (*n*>3). *P<0.01 (**c**) Caco-2 cells transduced with shControl and shPuma were transiently transfection of Bim-targeting small interfering RNA (siBim) or control siRNA (siNone). Silencing of both Bim and Puma was confirmed by western blotting. Cells were treated with 10-20 *μ*M RM4819 or 0.1% DMSO for 40 h. Cell death induction was assessed by an MTT assay. Mean values of triplicates±S.D. of a representative experiment are shown (*n*>3)

The profound increase in Bim and Puma expression levels suggested that these BH3-only proteins might represent critical components in the activation of the mitochondrial apoptosis pathway. To test this hypothesis Bim and Puma were knocked down using lentiviral small hairpin RNA constructs inducing RNA interference. Infection of both Caco-2 and LS174T cells with specific lentiviruses resulted in a profound decrease in Bim, resp. Puma expression (Figures 3a and b). Control cells and cells with reduced Bim, resp. Puma expression were then stimulated with the thiazolides RM4819 and compound 2, or cisplatin as control. As expected compound 2 failed to induce cell death, whereas RM4819 induced a strong increase in apoptosis. Knockdown of both Bim and Puma induced a significant reduction of RM4819-induced cell death in Caco-2 and LS174T cells. Similarly, cisplatin-induced cell death was also attenuated in cells with downregulated Bim and Puma expression (Figures 3a and b), in agreement with previous observations.^36^

Since neither downregulation of Bim nor Puma alone completely inhibited RM4819-induced cell death, and BH3-only proteins often act in concert, we aimed at investigating whether combined downregulation of Bim and Puma resulted in a more pronounced inhibition of cell death. Control shRNA- or Puma shRNA-infected Caco-2 cells were transfected with control siRNA or siRNA against Bim. Knockdown efficiency for these two BH3-only proteins was confirmed by western blot and revealed efficient silencing of Bim and Puma (Figure 3c). When cells were stimulated with increasing concentrations of RM4819 a partial protection was seen in Bim and Puma single knockdown cells. Importantly, combined knockdown of both BH3-only further increased protection from RM4819-induced cell death (Figure 3c), indicating that Puma and Bim cooperate in inducing the mitochondrial apoptosis pathway.

### Bim and Puma interact with pro-survival Bcl-2 members

**Figure 4:**
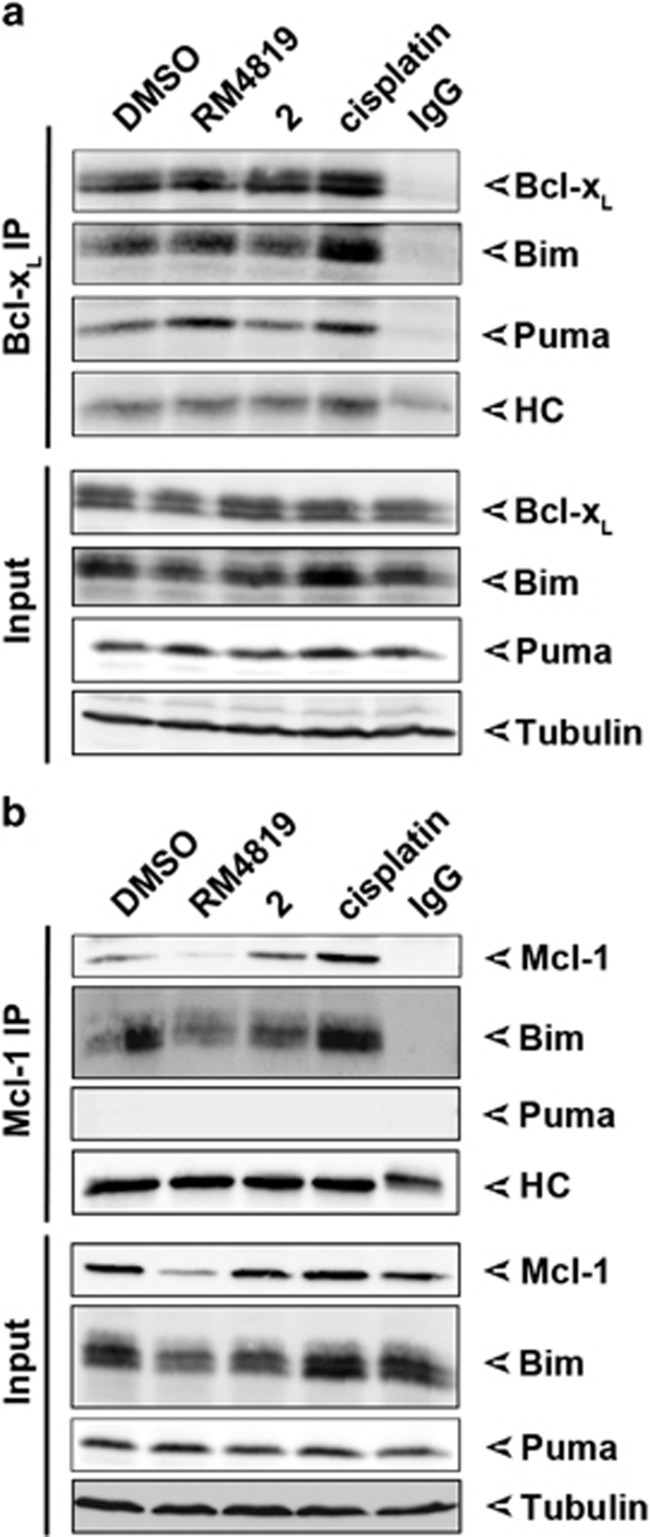
RM4819-induced binding of Bim and Puma to Bcl-x_L_ and Mcl-1. Caco-2 cells were treated with 0.1% DMSO, 20 *μ*M RM4819 or compund 2, or 10 *μ*g/ml cisplatin for 8 h. (**a** and **b)** Bcl-x_L_ (**a**) and Mcl-1 (**b**) were immunoprecipitated and interaction with Bim and Puma was confirmed by western blotting. In input controls 4% of the total lysates, resp. 8% of the precipitates were loaded. IgG, isotype control; HC, immunoglobulin heavy chain

As at least one mechanism how BH3-only proteins promote apoptosis is the binding to and neutralization of anti-apoptotic Bcl-2 members,^37^ we addressed the question whether thiazolides promote Bim and Puma binding to Bcl-x_L_ and Mcl-1. When Bcl-x_L_ was immunoprecipitated a constitutive binding of Bim and Puma was observed. Treatment of cells with RM4819 and cisplatin seemed to further induce Bim binding to Bcl-x_L_, whereas compound 2 failed to do so (Figure 4a). More pronounced were the changes in the binding of Puma to Bcl-x_L_. While only low levels of Puma were bound to Bcl-x_L_ in control, cisplatin- or compound 2-treated cells, stimulation of cells with RM4819 resulted in a strong increase in Puma binding to Bcl-x_L_. This is likely due to the RM4819-induced increase in Puma expression (Figures 2a and 4a,Supplementary Figure 2). When BH3-only binding to Mcl-1 was analyzed only minimal changes in Bim binding were observed. In RM4819-stimulated cells even a reduced co-immunoprecipitation of Bim was observed, likely due to the fact that already lower levels of Bim were evident in cell lysates at this time point after RM4819 stimulation (8 h), and Bim might already being consumed and degraded during the process of apoptosis induction. Furthermore, RM4819 also resulted in the degradation of Mcl-1. In contrast, while Puma was detectable in cell lysates and induced by RM4819, no Puma binding to Mcl-1 was detected in these co-immunoprecipitation experiments (Figure 4b). Thus, RM4819-induced Puma appears to interact predominantly with Bcl-x_L_.

### JNK mediates Bim and Puma activation

**Figure 5:**
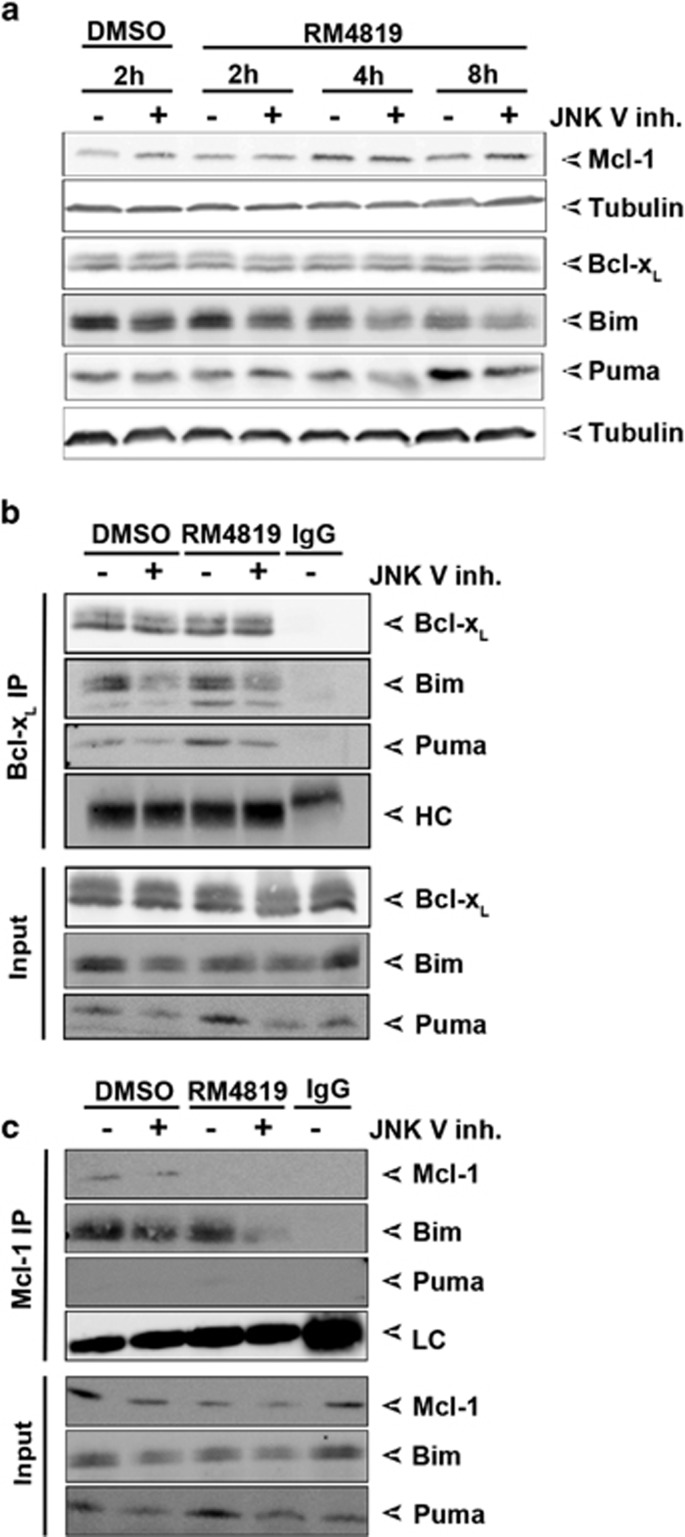
JNK mediates Bim and Puma induction and activation. (**a**) Caco-2 cells were pretreated with buffer control or JNK V inhibitor (2.5 *μ*M) for 1 h before treatment with 0.1% DMSO or 20 *μ*M RM4819 for 2, 4 and 8 h. Mcl-1, Bcl-x_L_, Bim, and Puma were detected by western blot. Tubulin served as a loading control. (**b** and **c**) Caco-2 cells were pretreated with JNK V inhibitor for 1 h, before stimulation with RM4819 or DMSO for 8 h. Bcl-x_L_ (**b**) and Mcl-1 (**c**) were immunoprecipitated, and Puma and Bim were detected by western blotting. IgG, isotype control; HC, immunoglobulin heavy chain; LC, immunoglobulin light chain

As the MAP kinases p38 and JNK have been found to be critically involved in thiazolide-induced apoptosis in colorectal tumor cells (Figures 1c and e),^31^ we next aimed at analyzing their role in Bim and Puma induction and activation. Cells were thus treated with RM4819 for different time points in the presence or absence of JNK inhibitor. The presence of JNK inhibitor appeared to slightly stabilize Mcl-1 levels in untreated cells, but only minimally prevent its thiazolide-induced degradation, whereas Bcl-x_L_ levels remained unaffected (Figure 5a and Supplementary Figure 3). It also had a profound effect on thiazolide-induced Bim and Puma levels. JNK inhibition resulted in decreased Bim levels in both control treated and RM4819-treated cells, and inhibited RM4819-induced Puma induction (Figure 5a and Supplementary Figure 3). Inhibition of p38 had less pronounced effects on Bim and Puma expression. Only minimally reduced Bim or Puma levels were seen in cells treated with p38 inhibitor and RM4819 (Supplementary Figure 4). Thus, Bim and Puma expression appears to be regulated predominantly by JNK.

We next assessed how JNK inhibition would affect RM4819-induced activation and binding of Bim and Puma to Bcl-x_L_ and Mcl-1. Even more pronounced than its effect on Bim expression was the JNK inhibitor-induced inhibition of Bim binding to Bcl-x_L_, in both control and RM4819-stimulated cells. Whereas Puma inducibly bound to Bcl-x_L_ inhibition of JNK strongly attenuated Puma binding. As seen before no Puma binding to Mcl-1 could be detected, whereas Bim binding to Mcl-1 was also reduced upon inhibition of JNK (Figure 5c). This indicates that JNK has an important role in the regulation of Bim and Puma expression, and their activation and binding to anti-apoptotic Bcl-2 family members.

### Increase in intracellular GSH levels enhances thiazolide-induced JNK activation and apoptosis induction

**Figure 6:**
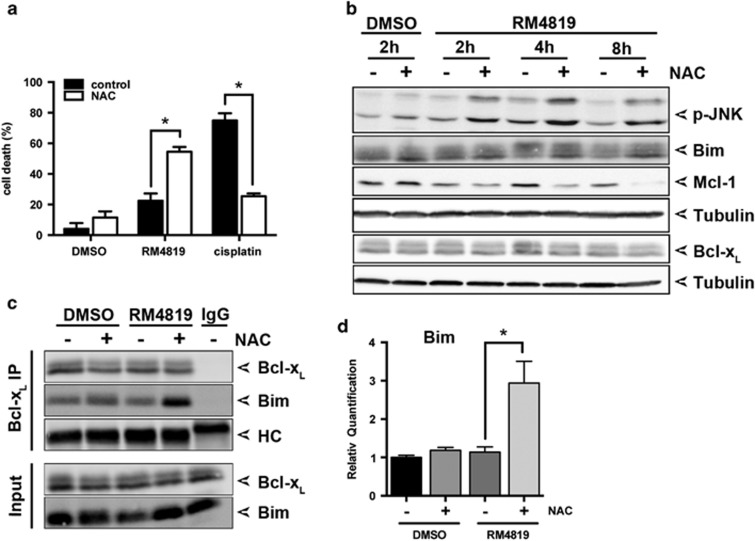
Increased GSH levels promote thiazolide-induced apoptosis. (**a**) Caco-2 cells were pretreated with 10 mM NAC for 1 h, before stimulation with RM4819 (10 *μ*M), cisplatin (10 *μ*g/ml), or DMSO (0.1%) for 40 h. Cell death induction was monitored by an MTT assay. Mean values of triplicates±S.D. of a representative experiment are shown (*n*>3). *P<0.0001. (**b**) Caco-2 cells were pretreated with 10 mM NAC for 1 h, followed by stimulation with 0.1% DMSO or 20 *μ*M RM4819 for times indicated. Phosphorylated JNK (p-JNK), Bim, Mcl-1, Bcl-x_L_, and tubulin were detected by western blot. (**c**) Caco-2 cells were pretreated with 10 mM NAC for 1 h, and stimulated with 20 *μ*M RM4819 or 0.1% DMSO for 8 h. Interaction of Bim with Bcl-x_L_ was assessed by immunoprecipitation and western blotting. IgG, isotype control; HC, immunoglobulin heavy chain. (**d**) Caco-2 cells were pretreated with 10 mM NAC for 1 h, and stimulated with DMSO (0.1%) or RM4919 (20 *μ*M) for 8 h. Bim mRNA expression was assessed by quantitative RT-PCR. Mean values of experimental triplicates±S.D. of a representative experiment are shown (*n*>3). *P<0.01. RQ, relative quantification

GSH is an important anti-oxidant in cells. GSTs couple GSH to xenobiotics and drugs, and thereby inactivate them.^1, 2^ NAC is a precursor of GSH, and treatment of cells with NAC leads to increased intracellular GSH levels.^31^ We have been previously suggesting that GSTP1-1 may also couple GSH to the pro-drug thiazolides, thereby generating a bioactive and pro-apoptotic product, since treatment of Caco-2 cells with NAC sensitized them to thiazolides, yet reduced the sensitivity to cisplatin.^31^ This observation was confirmed in Figure 6a, demonstrating enhanced thiazolide-induced cell death in NAC-treated cells. We thus set to analyze the effect of NAC on JNK activation, and Bim and Puma induction and activation. When cells were treated with NAC and stimulated with RM4819 an even more pronounced degradation of Mcl-1 was observed, whereas levels of Bcl-x_L_ remained unaffected (Figure 6b). Along these lines a strongly enhanced and sustained activation of JNK was observed. In contrast, only a minimal increase in p38 activation was observed (Supplementary Figure 5b). Next the effect of NAC on Bim expression and binding to Bcl-x_L_ was assessed. NAC treatment appeared to induce an increase in Bim protein levels, and contribute also to the stabilization of Bim protein expression after RM4819 treatment (Figures 6b and c). Along these lines, a profound increase in Bim binding to Bcl-x_L_ was observed (Figure 6c), suggesting also an increase in Bim activation. The relative NAC-mediated increase in Bim expression was also confirmed on mRNA level, where co-treatment with NAC and RM4819 leads to a threefold increase in Bim expression (Figure 6d).

In contrast to Bim, no increase or rather a slight decrease in Puma expression was seen, although JNK activation was drastically increased (Supplementary Figures 5b and c), and a contribution of JNK activity in the RM4819-induced Puma expression has been shown above (Figure 5a). This may be possibly explained by the antioxidant activity of NAC. Treatment of cells with RM4819 leads to increase in intracellular reactive oxygen species (ROS) (Supplementary Figure 5a), which may also contribute to Puma induction, possible via induction of DNA damage. ROS are, however, efficiently scavenged by NAC and NAC-induced increased levels of GSH. In line with this hypothesis, cisplatin-induced JNK and p38 activation, known to be induced via ROS,^38^ was also attenuated by NAC (Supplementary Fig. 5c).

## Discussion

Current cancer therapy often targets specifically survival- and cell death-regulating processes,^39^ for example by using chemotherapeutic drugs. GSTs have an important function in the detoxification of cells in general, and the detoxification of tumor cells from such chemotherapeutic drugs in particular.^1, 2, 8, 9^ Coupling of drugs to GSH inactivates and targets them for rapid secretion via bile or kidneys. Furthermore, more recently GSTs have also been found to sequester MAP kinases and thereby affect cellular signaling processes, such as apoptosis induction.^40^ It is thus not surprising that GSTs are overexpressed in a large number of tumors. Specifically, GSTP1-1 levels are increased in ovarian, lung, breast, kidney, pancreas, and colon cancer, and lymphomas,^10, 11, 12^ and limit chemotherapy.^13, 14, 15, 16, 17^

Given the important function of GSTs in detoxification and tumor cell survival, GSTs have become important therapeutic drug targets. EA is a potent GST inhibitor.^18, 41, 42^ However, clinical trials have not been successful due to its massive diuretic effects.^19^ TLK199 is another GST-activated GSTP1-1 inhibitor, which is currently in different clinical trials for the treatment of myelodysplatic syndrome, with a similar mode of action, that is, as a GSH-activated GST inhibitor.^20^ Various aspects indicate that the here described thiazolides act differently. Although we have initially seen that thiazolides bind to GSTP1-1 and inhibit the enzymatic activity of GSTP1-1 *in vitro*, very likely this observation was based on substrate competition. Indeed, we have seen later that enzymatic GSTP1-1 activity is required for thiazolides to kill colorectal tumor cells, and that increased GSH levels enhance thiazolide activity^31, 32^ (Figure 6a). Thus, very likely GSTP1-1 couples GSH to thiazolides, and thereby generates active apoptosis-promoting products. Furthermore, thiazolides sensitize tumor cells to triggers of the mitochondrial (chemotherapeutic drugs) and the extrinsic pathway (TRAIL).^31^ While high GSTP1-1 expression makes them very resistant to chemotherapeutic drugs and metabolites, it renders the cells also more susceptible to thiazolides. Thus, thiazolides bypass an important tumor resistance mechanism.^31^ Last, but not least, thiazolides are already successfully used in the clinic for the treatment of intestinal infections^24, 25, 26, 27, 28^ and show very little side effects on normal tissue cells. Given that thiazolides are well tolerated, and sensitize tumor cells to chemotherapeutic drugs, the combined use of thiazolides with other anti-cancer drugs may also allow to administer lower drug doses with similar anti-tumor efficacy but reduced side effects on tissue cells.

Though it is clear that GSTP1-1 converts the pro-drug RM4819 to an active component, thus far the underlying molecular processes causing cell death were not understood. Here we now show that exposure of cells to thiazolides induces MAPK activation, and that MAP kinase activation is important for the execution of thiazolide-induced cell death. Combined activation of JNK and p38 appears to promote the transcriptional induction and possibly also activation of the BH3-only proteins Bim and Puma. These two proteins bind and neutralize Bcl-x_L_ and Mcl-1, thereby promoting apoptosis. Thiazolides not only induce neutralization of Bcl-x_L_ and Mcl-1, but also promote Mcl-1 degradation, which likely further lowers the apoptosis resistance of tumor cells.

The induction and phosphorylation-induced activation of Bim is likely directly mediated by JNK. JNK inhibition results in reduced expression of Bim and reduced binding to Mcl-1. When cells were stimulated with active thiazolides, but not with inactive thiazolides, a shift of Bim to higher molecular weight forms is observed, likely representing JNK-mediated Bim phosphorylation (Figure 2a). Thus, JNK appears to regulate Bim at the transcriptional and post-translational level. Also thiazolide-induced Puma expression is likely controlled by JNK, as inhibition of JNK leads to reduced thiazolide-induced expression levels of Puma (Figure 5a) and reduced Puma binding to Bcl-x_L_ (Figure 5b). At present the mechanism of thiazolide-induced MAP kinase activation is unclear; however, the recently described pathway, in which GSTP1-1 sequesters and inactivates JNK,^40^ which may be reversed by thiazolides, has to be explored in further detail.

While thiazolides are not extremely potent pro-apoptotic drugs, they potently synergize with other apoptosis triggers, such as TRAIL and cisplatin.^31^ They appear to bring cells into a sensitized stage where little activation by other triggers is required to cause massive apoptosis induction.^31^ Thus, thiazolides may be useful for future combination therapy with conventional chemotherapeutic drugs. In addition, thiazolides alone are well tolerated by tissue cells, possibly because not all cells have high GSTP1-1 levels, promoting the activation of thiazolides. Furthermore, we have previously made the intriguing observation that thiazolides are only active in proliferating cells,^30^ although the underlying mechanism is presently not understood. Yet, the combination of restricted GSTP1-1 expression and proliferation status may protect tissue cells and bone marrow cells from thiazolide-induced destruction, while it renders tumor cells, specifically colorectal tumor cells, highly sensitive to thiazolide-induced apoptosis. Though clinical trials have to prove this concept and the overall usefulness of thiazolides in the treatment of colorectal tumors, the present data suggest that thiazolides may represent an interesting novel therapeutic approach for the treatment of this tumor entity.

## Materials and Methods

### Cell lines and reagents

The colon cancer cell lines Caco-2 (ATCC HTB-37) and LS174T (ATCC CL-188) were obtained from American Type Culture Collection (ATCC) and cultured in Iscove's Modified Dulbecco's Medium (IMDM) supplemented with 10% fetal calf serum (FCS), 4 mM L-glutamine, and 50 *μ*g/ml gentamicin (all from Sigma-Aldrich, Steinheim, Germany). Cells were cultured at 37 °C and 5% CO_2_ in a humidified atmosphere. Cisplatin and 3-(4,5-dimethylthiazol-2-yl)-2,5-diphenyltetrazoliumbromid (MTT) were obtained from Sigma-Aldrich and DMSO from ROTH (Karlsruhe, Germany). JNK V inhibitor and p38 inhibitor SB202190 were purchased from Calbiochem (Darmstadt, Germany), the dye 6-carboxy-2′, 7′-dichlorodihydrofluorescein diacetate (carboxy-H2DCFDA) from Life Technologies (Darmstadt, Germany). Thiazolides RM4819 and compound 2 were synthesized in-house as described earlier.^32^ Compounds were kept as 20 mM stock solutions in DMSO.

### MTT assay

Cell viability was measured by using an MTT assay. Caco-2 and LS174T cells were seeded into 96-well plates at 5–8 × 10^3^ cells/well. After overnight attachment, cells were treated with thiazolides, cisplatin, or DMSO as a control for 40 h. In some experiments JNK and/or p38 inhibitors were added 1 h before apoptosis induction. In some assays cells were treated with 10 mM *N*-acetyl-L-cysteine (NAC, Sigma-Aldrich) for 1 h to increase intracellular GSH levels. At the end of the experiment cell culture medium was discarded and replaced with 0.5 mg/ml MTT solution, dissolved in complete medium. Plates were incubated under cell culture condition for an additional 1-2 h. After MTT reduction to purple formazan, the MTT solution was discarded and replaced with 100 *μ*l DMSO to dissolve the formazan products. Plates were incubated for 15 min in a dark box at room temperature. After gently mixing of the plates, the intensity of the colored solution was quantified by measuring the absorbance at *λ*=562 nm on an ELISA reader (Tecan, Crailsheim, Germany). Cell death induction (%) was calculated as 100 × (1− (OD exp. mean value (−substrate blank)/OD control mean value (− substrate blank)).

### Bim and Puma knockdown in Caco-2 and LS174T cells using lentiviruses

HEK293T cells were seeded into 10 cm petri-dish and co-transfected with the packaging plasmid pCMVdeltaR8.2, the envelope plasmid pMD2-VSV-G and pLKO-based plasmid containing shRNA against human Bim (NM_138621.X-541s1c1 and NM_138621.3-559s21c1) and Puma (NM_014417.2-1318s1c1 and NM_014417.2-785s1c1) (pMission, Sigma-Aldrich) using Roti®-Fect Plus (ROTH). Plasmid SHC002 (Sigma-Aldrich) served as non-target control. After 24 h, medium was changed with complete Dulbecco's Modefied Eagle's Medium (DMEM) containing 10% FCS and 50 *μ*g/ml gentamicin for further 24 h. In parallel, Caco-2 and LS174T were seeded into six-well plates (1.8–2.2 × 10^5^ cells/well). Next day, viruses in the supernatant were collected and filtered through a 0.45 *μ*m filter and charged with 8 *μ*g/ml polybrene (Sigma-Aldrich). Target cells were then transduced with the virus mixture in a 1 : 2 dilution for 1 day. Stable cells were selected using 3 *μ*g/ml puromycin. Silencing of Bim and Puma was verified by western blotting.

### Double knockdown of Bim and Puma in Caco-2 cells

For double knockdown experiments, Bim expression in control shRNA or Puma shRNA-transduced Caco-2 cells was silenced using Bim siRNA (L-004383-02) or control siRNA (D-001820-01) (Dharmacon, siGenome SMARTpool). The cells were seeded into six-well plates (1.8 × 10^6^) and transfected with siRNAs for 24 h using Lipofectamin®2000 (Life Technologies, Carlsbad, CA) as transfection reagent, as done previously.^31, 34^ Afterward, cells were transferred to a 96-well plate (5 × 10^3^ cells/well) and used for cytotoxicity assays. In parallel, Bim and Puma knockdown was verified 48 h post transfection by western blot.

### Western blot

Caco-2 or LS174T cells were seeded into 10 cm petri-dish (2 × 10^6^) and treated with thiazolides (20 *μ*M), cisplatin (10 *μ*g/ml), DMSO (0.1%), or with complete medium as control for 0, 2, 4, and 6 h and additional 8 h. In some experiments, Caco-2 cells were pretreated with JNK inhibitor (2.5 *μ*M) or with p38 inhibitor (10 *μ*M) or with NAC (10 mM) for 1 h. before treatment with RM4819, cisplatin, or DMSO for up to 16 h. Cells were then lysed in NP40-lysis buffer (150 mM NaCl, 50 mM Tris pH 7.6, 1 mM EDTA and 1% NP-40) and cell lysates were separated on a denaturing 12% SDS-PAGE gel. After transfer to polyvinylidene difluoride membranes (PVDF) (Roche, Mannheim, Germany), Mcl-1, Bcl-x_L_, Bim, Puma, phospho-JNK, phospho-p38, or tubulin as loading control were detected using specific antibodies (anti-Mcl-1 from eBioscience (Frankfurt, Germany); anti-Bcl-x_L_ (54H6), anti phospho-SAPK/JNK (Thr183/Tyr185) (81E11), anti-phopho–p38 MAPK (Thr180/Tyr182) (D3F9), anti-Puma (D30C10) from Cell Signaling Technology (Davers, MA, USA); anti-Bim, anti-*α*-Tubulin from Sigma-Aldrich). Proteins were detected using horse radish-coupled secondary antibodies and enhanced chemiluminescence on an Image Quant LAS 4000 (GE Healthcare, Braunschweig, Germany).

### Immunoprecipitation

Caco-2 cells were seeded into 10-cm petri dishes (2 × 10^6^ cell/well) and treated with thiazolides (20 *μ*M), cisplatin (10 *μ*g/ml), or DMSO (0.1%) for 8 h. In some experiments cells were additionally pretreated with 2.5 *μ*M JNK inhibitor or 10 mM NAC for 1 h. Cells were then harvested by trypsinization, washed once with PBS and lysed using 500 *μ*l NP-40 lysis buffer containing a protease inhibitor cocktail. Cell lysates were then precleared using Sepharose protein G beads (GE Healthcare, Freiburg, Germany), and Mcl-1 and Bcl-x_L_ were immunoprecipitated using Sepharose protein G beads coupled with specific antibodies or isotype controls for 24 h at 4 °C under constant agitation. After washing proteins were eluted by boiling in SDS-PAGE loading buffer and resolved on a 12% SDS-PAGE. Proteins detected by western blot as described above.

### Detection of mRNA expression

Caco-2 cells were seeded into 10-cm petri dishes (2 × 10^6^ cell/well). After attachment, cells were pretreated with 10 mM NAC for 1 h to increase the GSH level before treatment with RM4819 (20 *μ*M) or DMSO (0.1%) for further 8 h. Cells were harvested, lysed in PeqGOLD TriFast (PeqLab, Erlangen, Germany) RNA isolation reagent and RNA was isolated according to the manufacturer's protocol. One microgram of RNA was reverse transcribed and cDNAs were used for quantification of gene expression by quantitative PCR using FASTSYBR Green Master Kit and a StepOnePlus Real time PCR system (Applied Biosystems, Foster City, CA, USA). The following primers were used: human Bim forward 5′-ATG AGA AGA TCC TCC CTG CT-3′ and reverse 5′-AAT GCA TTC TCC ACA CCA GG-3′, GAPDH forward 5′-ATG GAG AAG GCT GGG GCT CA-3′ and reverse 5′-AGT GAT GGC ATG GAC TGT GGT CAT-3′ to normalize the gene expression by GAPDH expression levels.

### Reactive oxygen species (ROS) measurement

Caco-2 cells were incubated with 10 *μ*M H2DCFDA for 1 h, before stimulation with 20 *μ*M thiazolides, 1 mM H_2_O_2,_ or 0.1% DMSO for 15 min. In the presence of ROS, the H2DCFDA molecule becomes oxidized and fluorescents at Ex/Em: ~492–495/517–527 nm. The mean fluorescence intensity (MFI) was analyzed by flow cytometry on a LSR Fortessa Cell Analyzer using FACS Diva software (BD Biosciences, Heidelberg, Germany).

### Statistics

Statistical differences were analyzed using Prism 5 software and a multiple *t*-test. For experiments described in Supplementary Figure 4a, a one-way ANOVA with Dunnett's multiple comparison post-test was performed.

## Abbreviations

Ctrl: control
GSTP1-1: glutathione-*S*-transferase pi 1
GSH: glutathione
HC: heavy chain
IgG: immunoglobulin G
LC: light chain
MAP kinases: mitogen-activated protein kinases
MTT: 3-(4,5-dimethylthiazol-2-yl)-2,5-diphenyltetrazoliumbromid
carboxy-H2DCFDA: 6-carboxy-2′,7′-dichlorodihydrofluorescein diacetate
NAC: *N*-acetyl-l-cysteine
Qpcr: quantitative polymerase chain reaction
RT-PCR: reverse transcriptase polymerase chain reaction
