# Supplementary material for: Thiazolides promote apoptosis in colorectal tumor cells via MAP kinase-induced Bim and Puma activation
Source: Cell Death Dis. 2015 Jun 4;6(6):e1778–. doi: 10.1038/cddis.2015.137 (PMC4669824; doi:10.1038/cddis.2015.137)
Supplement: Supplementary Figure 1 [file cddis2015137x1.pdf]

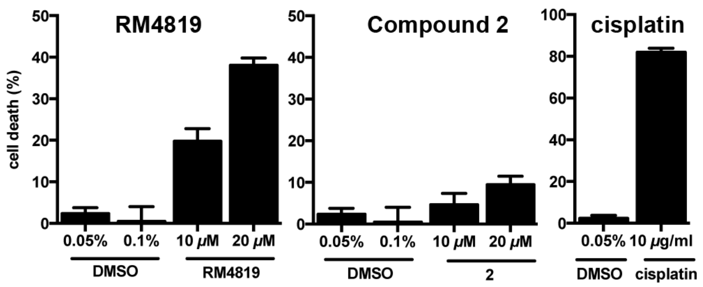

Supplementary Figure 1: *Thiazolide-induced cell death in LS174T cells.* LS174T cells were stimulated with 10-20 *µ*M RM4819 or compound 2, or corresponding concentrations of DMSO, for 40h. Cisplatin (10 *µ*g/mL) was as a positive control. Cell death induction was monitored by MTT assay. Mean values of triplicates ± SD of a representative experiment are shown (n > 3).
