# Supplementary material for: Thiazolides promote apoptosis in colorectal tumor cells via MAP kinase-induced Bim and Puma activation
Source: Cell Death Dis. 2015 Jun 4;6(6):e1778–. doi: 10.1038/cddis.2015.137 (PMC4669824; doi:10.1038/cddis.2015.137)
Supplement: Supplementary Figure 2 [file cddis2015137x2.pdf]

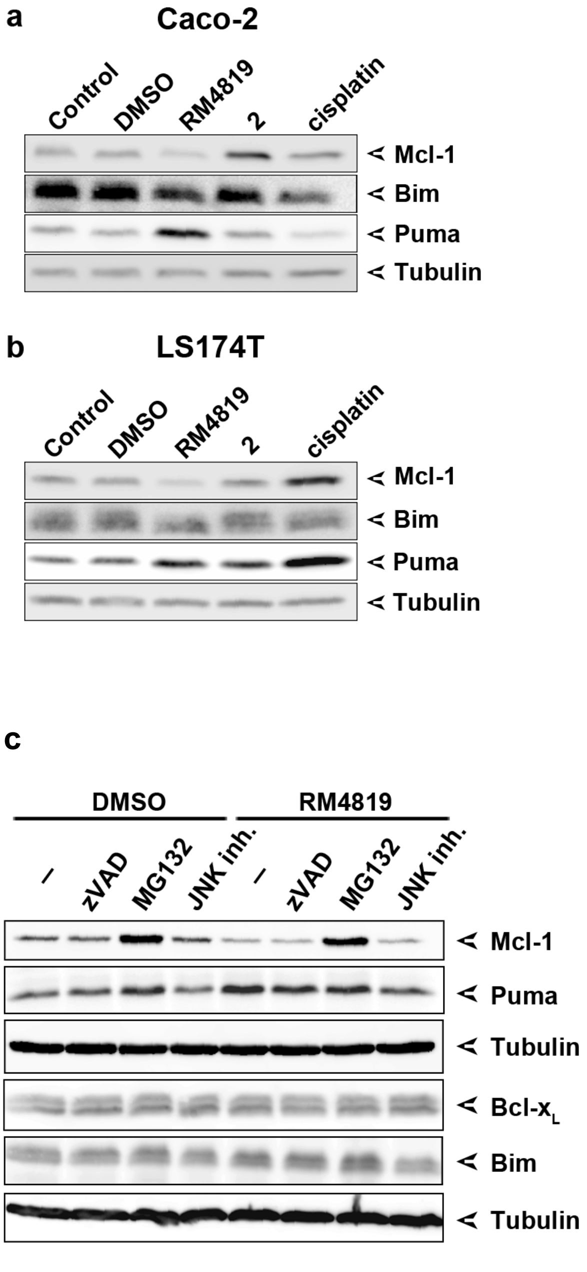

Supplementary Figure 2: *Thiazolide-induced changes in Mcl-1, Bim and Puma expression.* Caco-2 **(a)** and LS174T cells **(b)** were treated with complete medium, 0.1% DMSO, 20 μM RM4819 or compound 2 or 10 μg/mL cisplatin for 16 h. Mcl-1, Bim and Puma were monitored by Western blotting. Tubulin served as loading control. c) Caco2 cells were pretreated with control, the caspase inhibitor zVAD (80 μM), proteosome inhibitor MG132 (10 μM) or with JNK V inhibitor (2.5 μM), prior to stimulation with DMSO control or 20 μM RM4819 for 8 h. Mcl-1, Bcl-x$_L$, Bim and Puma were detected by Western Blot. Tubulin served as loading control.
