# Supplementary material for: Thiazolides promote apoptosis in colorectal tumor cells via MAP kinase-induced Bim and Puma activation
Source: Cell Death Dis. 2015 Jun 4;6(6):e1778–. doi: 10.1038/cddis.2015.137 (PMC4669824; doi:10.1038/cddis.2015.137)
Supplement: Supplementary Figure 3 [file cddis2015137x3.pdf]

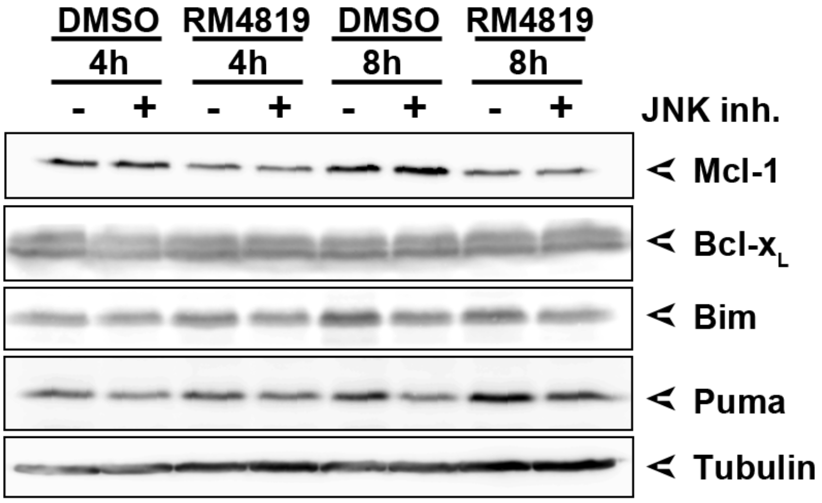

Supplementary Figure 3: Effect of JNK inhibition on Bcl-2 family member protein levels in control and RM4819-treated cells

Caco-2 cells were pretreated with buffer control or JNK V inhibitor (2.5 μM) for 1 h. Afterward, cells were treated with 0.1% DMSO or 20 μM RM4819 for 4 h and 8 h. Mcl-1, Bcl-x_L, Bim and Puma were detected by Western Blot. Tubulin served as loading control.
