# Supplementary material for: Thiazolides promote apoptosis in colorectal tumor cells via MAP kinase-induced Bim and Puma activation
Source: Cell Death Dis. 2015 Jun 4;6(6):e1778–. doi: 10.1038/cddis.2015.137 (PMC4669824; doi:10.1038/cddis.2015.137)
Supplement: Supplementary Figure 4 [file cddis2015137x4.pdf]

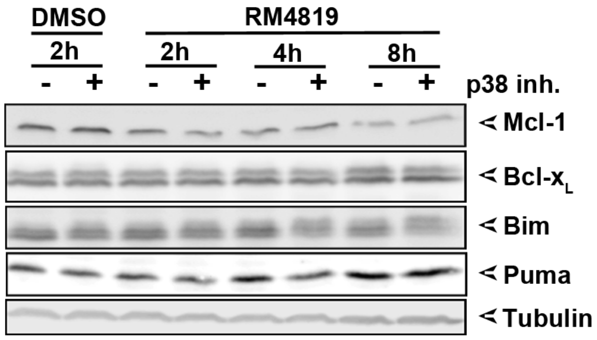

Supplementary Figure 4: *Role of p38 in thiazolide-mediated Bim and Puma induction.* Caco-2 cells were pretreated with 10 µM p38 inhibitor for 1 h, prior to stimulation with solvent control (0.1% DMSO), or RM4819 (20 µM) for times indicated. Mcl-1, Bcl-x_L, Bim and Puma were detected by Western blotting. Tubulin served as loading control.
