# Supplementary material for: Thiazolides promote apoptosis in colorectal tumor cells via MAP kinase-induced Bim and Puma activation
Source: Cell Death Dis. 2015 Jun 4;6(6):e1778–. doi: 10.1038/cddis.2015.137 (PMC4669824; doi:10.1038/cddis.2015.137)
Supplement: Supplementary Figure 5 [file cddis2015137x5.pdf]

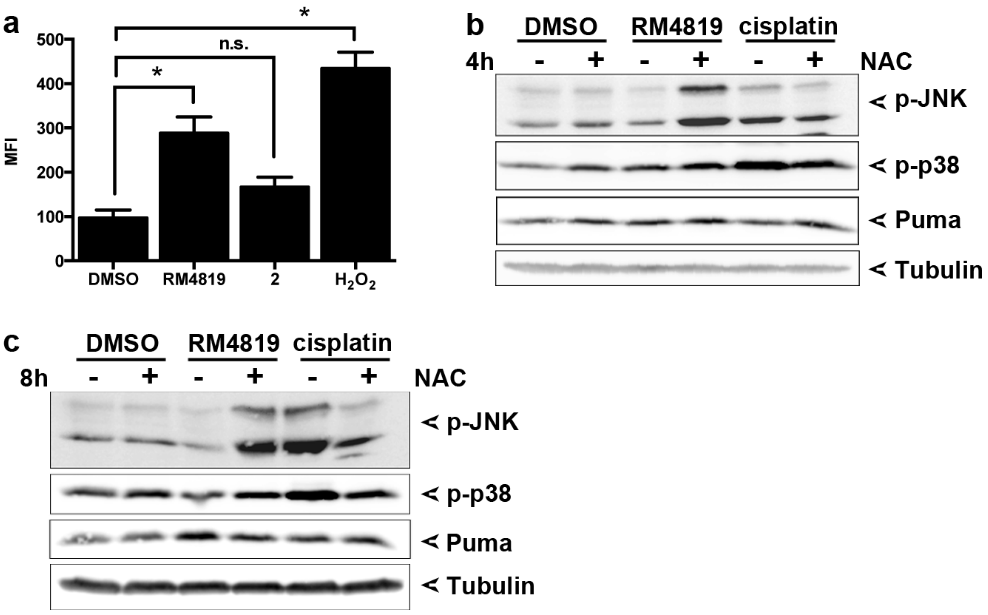


**Supplementary Figure 5:** *Increase in intracellular GSH levels enhances thiazolide-induced JNK activation and apoptosis induction*. **a)** Measurement of Reactive oxygen species (ROS). Caco-2 cells were loaded with the ROS-sensitive dye H2DCFDA (10 *µ*M) for 1 h before stimulation with 0.1% DMSO, 20 *µ*M RM4819 or compound 2, or 1 mM $H_2O_2$ for 15 min. The mean fluorescence intensity (MFI) was measured by flow cytometry. Mean values of triplicates ± SD of a typical experiment (n=3) are shown. Data were analyzed with a one-way ANOVA followed by Dunnett's multiple comparison post-test (*p < 0.0001; n.s., not significant) **b)** - **c)** Cells were pretreated with 10 mM NAC, and stimulated with DMSO (0.1%), RM4819 (20 *µ*M) or cisplatin (10 *µ*g/mL) for 4 h **(b)** and 8 h **(c)**. Phosphorylated JNK (p-JNK), phosphorylated p38 (p-p38) and Puma were monitored by Western blotting. Tubulin served as loading control.
